# Social-ecological factors associated with loneliness in university students: results from the German cross-sectional StudiBiFra study

**DOI:** 10.3389/fpsyt.2025.1469811

**Published:** 2025-07-07

**Authors:** Vanessa Wenig, Katherina Heinrichs, Eileen Heumann, Jennifer Lehnchen, Julia Burian, Zita Deptolla, Christiane Stock

**Affiliations:** ^1^ Charité – Universitätsmedizin Berlin, corporate member of Freie Universität Berlin and Humboldt- Universität zu Berlin, Institute of Health and Nursing Science, Berlin, Germany; ^2^ Health Management, Bielefeld University, Bielefeld, Germany; ^3^ Unit for Health Promotion Research, University of Southern Denmark, Esbjerg, Denmark

**Keywords:** loneliness, mental health, university students, social-ecological factors, social support, health promotion

## Abstract

**Aims:**

Using a social-ecological perspective, this study aims to understand loneliness in university students by (1) assessing its prevalence, and (2) identifying inter-personal and organizational factors associated with loneliness during studies.

**Methods:**

Data from the StudiBiFra study, a cross-sectional survey among university students in Germany, were used. The sample consisted of 12,874 students from 7 universities, surveyed between May 2022 and March 2023 using the Bielefeld Questionnaire on Study Conditions and Mental Health. Hierarchical logistic regression was employed to examine the associations of individual, inter-personal, and organizational variables with loneliness.

**Results:**

A total of 28.2% of students experienced loneliness during their studies. Gender-diverse students (OR = 1.69; 95% CI: 1.04 – 2.73) and males (OR = 1.43; 95% CI: 1.26 – 1.63), as well as those with poor subjective overall health (OR = 2.62; 95% CI: 2.26 – 3.05), were at high risk of feeling lonely. At the inter-personal level, positive social relationships among students acted as a protective factor against loneliness (OR = 0.31; 95% CI: 0.29 – 0.34). At the organizational level, weak connectedness to the university was positively associated with loneliness (OR = 1.43; 95% CI: 1.23 – 1.67), while high university engagement was negatively associated with loneliness (OR = 0.90; 95% CI: 0.83 – 0.97). Students enrolled in universities of applied sciences were less likely to experience loneliness compared to those at universities (OR = 0.76; 95% CI: 0.63 – 0.91).

**Conclusions:**

The findings underscore the importance of both individual and institutional efforts to address loneliness at German universities, a demand that was accelerated during and after the pandemic. Promoting social connections and strengthening students’ ties to their university are important strategies for combating loneliness, highlighting the importance of community-building health promotion.

## 1 Introduction

University students are at high risk of mental health problems such as depression, anxiety (1–3), and loneliness (3–6). In addition to the negative health effects of loneliness (7, 8), loneliness and mental health problems are also associated with poor academic outcomes for university students (9, 10). From a social-ecological perspective, loneliness is caused by a number of interrelated factors, including individual, inter-personal and environmental aspects (11). While it is crucial to identify predictors of loneliness, it is equally important to understand the variation in these risk factors across different contexts (12). In general, Bronfenbrenner’s social-ecological model of health is used to better understand and address health determinants on multiple interrelated levels (13). In this study, we refer to the social-ecological model framework for prevention (SEM) by the World Health Organization (WHO), originally proposed by Dahlberg et al. (14), with the Holt-Lundstad extension regarding social connectedness (11). Dahlberg et al. (14) distinguish four different levels: the individual level focuses on personal factors like age, education and several sociodemographic factors, with prevention strategies targeting attitudes and behaviors. At the inter-personal level, close social circles such as family and peers play a role, and prevention includes programs to improve communication and promote healthy relationships. At the community level, attention shifts to settings including schools and neighborhoods, with the aim of improving safety and addressing underlying conditions such as poverty. Finally, the societal level examines broader factors like cultural norms and societal inequalities, with prevention efforts targeting systemic issues, for instance economic inequality, and promoting norms (14). For the purposes of our study, we will only consider the first three levels of the SEM. We suggest that loneliness in university students is influenced by various factors at the individual, inter-personal, and organizational level. While previous research has focused on the individual contributions within these single domains, there has been minimal consideration of their combined impact.

Individual-level factors related to loneliness in university students have been well studied and are related to several sociodemographic characteristics: Previous research has shown that loneliness is associated with being single (4, 5, 15), living alone (4, 5, 16), or studying abroad (4, 5). Younger and older students are reported to feel more lonely than middle-aged students (4), and loneliness is linked to gender. Female (17, 18) and gender-diverse students (19) seemed to be particularly vulnerable to loneliness. Furthermore, loneliness among university students has been linked to mental health issues, depressive symptoms (5, 20, 21) and anxiety (5, 20), as well as a poor subjective health status (22).

The second level of the SEM examines inter-personal relationships, e.g., peer groups, partners, friends, or family, which may influence the experience of loneliness (14) and may also be stressors in academia (23). Social connection (or, conversely, social disconnection) is an umbrella term with components related to the structural aspects of social life (e.g., size of social network, living situation), the functional aspects of social life (e.g., received or perceived availability of social support), and the quality of social relationships (e.g., perceived satisfaction) (24). Together, these categories are associated with several (mental) health risks (25), and social connection is a significant protective factor for morbidity and mortality (24). In the university context, low levels of student cooperation, in terms of social support (22), deficits in friendships, and not feeling connected to peers were associated with loneliness among university students (26). However, university-based research is still limited. Prior studies of adolescents in schools showed that several social relationships were associated with loneliness: strong bonds with peers and face-to-face contact with friends (e.g., going out with friends) were protective factors against loneliness (12). Further, higher levels of peer victimization at school were associated with poorer mental health outcomes and increased loneliness (27, 28). In addition, previous research has highlighted perceived teacher behavior and peer cooperation as important determinants of loneliness. Higher levels of loneliness were associated with both lower levels of teacher support and lower levels of students’ cooperation (29).

The third level of the SEM explores the community or settings, such as schools and universities, in which social relationships take place, and attempts to identify the characteristics of these environments that contribute to people’s loneliness (14). However, university-based research on loneliness and organizational factors is also scarce. In general, previous research has shown that organizational factors, such as study conditions, may also be associated with mental health problems in students (30, 31). Similarly, socially supportive academic environments, feelings of belongingness regarding the sense of connectedness to learning environments, and inter-personal resilience are associated with better mental health outcomes and academic persistence (32). Previous school-based research has shown that social inclusion at school is a significant protective factor of adolescent loneliness, and that loneliness partially mediates the association of social inclusion with subjective well-being and mental health problems (33). Adolescents who had experienced social exclusion at school reported higher levels of loneliness (33). Some evidence also suggests that participating in school extracurricular activities and demonstrating high levels of school engagement can lead to higher levels of perceived social support and lower levels of perceived loneliness (34). In addition, loneliness at school is associated with poor academic performance and can lead to absenteeism due to health effects (35, 36).

Although research has examined geographical or regional characteristics associated with loneliness (37), or referred to the school context (27, 29), few studies have examined the prevalence of loneliness in the university context. However, there is an urgent need to develop effective methods and interventions that go beyond individual-level strategies (13). Using a social-ecological perspective, this study seeks to better understand the factors associated with loneliness in the university context. The aims of this study were [1] to assess the prevalence of loneliness in university students and, [2] to identify individual, inter-personal and organizational factors associated with loneliness.

Based on the current literature, we hypothesized that female and diverse gender as well as younger age and poor health state are associated with loneliness. Moreover, we assume that both student cooperation and lecturer support are positively associated with loneliness. We further suggest that positive experiences of the university culture, higher levels of university engagement, and a sense of connectedness to the university act as protective factors against loneliness.

## 2 Materials and methods

### 2.1 Study design and procedures

For the present study, we used data from the StudiBiFra project, a cross-sectional study aimed at assessing the study conditions and (mental) health status of students across thirteen German universities. The survey questionnaire (Bielefeld Questionnaire on Study Conditions and Mental Health) included questions on a range of topics including study conditions, academic outcomes, sociodemographic characteristics, and health-related outcomes. Further information on the survey questionnaire can be found elsewhere (38). Participants were able to complete it in either English or German. University students enrolled in undergraduate and postgraduate programs were invited by email. Data collection was conducted via LimeSurvey (LimeSurvey GmbH, Hamburg, Germany) and took place between June 2021 and March 2023. The StudiBiFra study has received ethical approval from the ethics committee of Charité - Universitätsmedizin Berlin (EA1/055/21). Written informed consent was obtained from all respondents prior to participation.

This analysis comprises data of seven universities. These institutions were selected because data collection occurred during a period devoid of strict COVID-19 protective measures. Data collection at the selected institutions took place between May 2022 and March 2023, and the average response rate obtained across these universities was 11.5%.

### 2.2 Measures

A more detailed description of the variables and their response options can be found in **Table 1**. For all items analyzed in this paper, students had the opportunity to choose the option ‘no answer’.

**Table 1:** Measurement of variables and response options.

Domain	Variables	Assessment	Response options
Dependent variable			
	Loneliness	‘I am lonely in my studies’	(1) ‘strongly disagree’ (2) ‘disagree’ (3) ‘neither disagree, nor agree’ (4) ‘agree’ (5) ‘strongly agree’
Independent variables			
Individual level	Gender	‘I identify with the following gender’	‘female’ ‘male’ ‘diverse’
	Age	‘I belong to the following age group’	‘18–25 years’ ‘26–30 years’ ‘31–40 years’ ‘41 and older’
	Overall health state	‘In the last two months of my studies, my overall state of health was’	(1) ‘severely affected’ (2) ‘rather affected’ (3) ‘neither affected, nor unaffected’ (4) ‘rather not affected’ (5) ‘not affected at all’
Inter-personal level	Social support among students	‘There are tensions and conflicts among the students’	(1) ‘strongly disagree’ (2) ‘disagree’ (3) ‘neither disagree, nor agree’ (4) ‘agree’ (5) ‘strongly agree’
		‘The (personal) relationships among the students in my course of studies are good’	
		‘In my course of studies, the students mutually support each other’	
		‘In my course of studies, it is easy to connect socially’	
	Lecturers’ support	‘My lecturers show appreciation for the individual students’	(1) ‘strongly disagree’ (2) ‘disagree’ (3) ‘neither disagree, nor agree’ (4) ‘agree’ (5) ‘strongly agree’
		‘My lecturers support cooperation among students (e.g., through group work)’	
		‘My lecturers are approachable in case of personal problems’	
		My lecturers are overall sufficiently available outside of the courses (e.g., office hours, email contact)’	
University level	University culture of open communication	‘Overall, people experience a culture of open communication at the university’	(1) ‘strongly disagree’ (2) ‘disagree’ (3) ‘neither disagree, nor agree’ (4) ‘agree’ (5) ‘strongly agree’
	University connectedness	‘I feel connected to my faculty’	(1) ‘strongly disagree’ (2) ‘disagree’ (3) ‘neither disagree, nor agree’ (4) ‘agree’ (5) ‘strongly agree’
	University engagement	‘I am committed to issues related to my course of studies (e.g., student council, committee work, teaching evaluations)’	(1) ‘strongly disagree’ (2) ‘disagree’ (3) ‘neither disagree, nor agree’ (4) ‘agree’ (5) ‘strongly agree’
		‘I make a contribution to the further development of my course of studies’	
		‘I am involved at my university (e.g., General Students’ Committee, university groups)’	

#### 2.2.1 Dependent variable: loneliness during studies

Consistent with other studies in this area (28, 39), our research objective focuses on ‘loneliness during studies’ rather than general loneliness. To examine the subjective perceptions of loneliness at university, participants were asked about their level of agreement with the statement: ‘I am lonely in my studies’ with five answering options: (1) ‘strongly disagree’ (2) ‘disagree’, (3) ‘neither disagree, nor agree’, (4) ‘agree’ (5) ‘strongly agree’. Those who strongly agreed or agreed with this statement (4 – 5) were grouped as ‘perceived loneliness during studies’ (reference group) and distinguished from those expressing neutrality or who (strongly) disagreed ‘no perceived loneliness during studies’ (1 – 3).

#### 2.2.2 Independent variables: individual level

At the individual level, we considered self-identification with gender, age, and overall self-perceived health state. The self-perceived health state was measured using the item: ‘In the last two months of my studies, my overall state of health was’. To facilitate comparative analysis among respondents, the responses were encoded into three distinct representations: those reporting being ‘severely’ or ‘rather affected’, those indicating neutrality (‘neither affected nor unaffected’), and those reporting no impact (‘rather not affected’ or ‘not affected at all’).

#### 2.2.3 Independent variables: inter-personal level

At the inter-personal level, we distinguish between the quality of students’ cooperation and the perceived support from lecturers. To assess social support among students, we used four items. For analysis, the first item was reversed, and sum and mean scores were calculated, ranging from 1 to 5, with a higher score indicating a higher level of perceived social support among students. Perceived lecturers’ support was measured by four items, and we used the same response options and mean calculation as for social support among students. The Cronbach’s alpha in our sample was α = 0.80 for student social support and α = 0.72 for lecturer support.

#### 2.2.4 Independent variables: university level (organizational level)

At the university level, we differentiated between university characteristics and students’ experience and engagement in university. University culture of open communication was assessed using one item. We grouped those who (strongly) agreed (4 – 5) with the statement, distinguished from those who were neutral (3) and those who (strongly) disagreed (1 – 2). The university connectedness was measured using one item with responses categorized according to the ‘university culture of open communication’. To assess university engagement, we used three items. These three items were summed to give an overall score, then the mean was calculated, ranging from 1 to 5, with a higher score indicating a higher level of university engagement. The Cronbach’s alpha in our sample was α = 0.72 for the university engagement scale. Further, we considered the type of university. In our study, we included students from universities and from universities of applied sciences as two distinct types of higher education institutions in Germany.

### 2.3 Data analyses

First, descriptive statistics were calculated for both sociodemographic characteristics and the prevalence of loneliness during studies. The prevalence of loneliness during studies in this sample was estimated based on the extent of agreement with the respective statement, as those who (strongly) agreed to feel lonely during their studies. The varying sample sizes in the descriptive calculations are due to participants having the option to choose ‘no answer’ for each item, and missing data were excluded. Second, a hierarchical approach was used to sequentially add the social-ecological domains of interest and examine the associations with loneliness as the dependent variable. Thus, the variables were added blockwise. Model 1 tested for associations between individual factors and loneliness. Inter-personal and university factors were added to models 2 and 3, respectively. Hierarchical regression was used to allow for a theoretically driven process whereby subsequent variables were incorporated into the model based on key research considerations (e.g., adding additional social-ecological domains of influence). Complete case analysis was utilized in the regression models. Prior to including the independent variables into the models, multicollinearity was assessed. The correlations among independent variables were found to be minimal (r <.70; VIF coefficients > 1.00 and < 5.00), suggesting that multicollinearity did not significantly impact the analysis. Results from the hierarchical logistic regression analyses were reported as odds ratios (ORs) and 95 percent confidence intervals (CIs). Statistical analyses were conducted using IBM SPSS, version 28.0.

## 3 Results

The characteristics of the study sample are presented in **Table 2**. Most participants were female (64.1%), with 1.6% identifying as gender-diverse. The majority of participants were aged between 18 and 25 years (71.5%) and studied at a university (84.7%), rather than a university of applied sciences (15.3%). In relation to their overall health state, 38.8% of students reported being (rather) unaffected, whereas 35.3% indicated being severely or rather affected. The culture of open communication of their university was perceived positively by most students (65.1%). However, almost half of students (43.6%) did not feel connected to their university. The perceived level of social support among students was slightly higher than the level of lecturers’ support.

**Table 2:** Characteristics of the sample (n = 12,874).

Variables	n	%	Mean	SD
Gender (n = 8,369)				
Female	5,361	64.1		
Male	2,874	34.3		
Diverse	134	1.6		
Age (n = 8,449)				
18 – 25	6,037	71.5		
26 – 30	1,657	19.6		
31 – 40	604	7.1		
41 and older	151	1.8		
Overall health state (n = 8,329)				
(Rather) not affected	3,230	38.8		
Neither affected nor unaffected	2,162	26.0		
Severely/rather affected	2,937	35.3		
Type of university (n = 12,874)				
University	10,908	84.7		
University of applied sciences	1,966	15.3		
University culture of open communication (n = 7,430)				
Positive perception	4,837	65.1		
Neutral perception	1,807	24.3		
Negative perception	786	10.6		
University connectedness (n = 8,201)				
(Strong) connectedness	2,765	33.7		
Neutral connectedness	1,864	22.7		
Weak connectedness	3,572	43.6		
Social support among students	8,585		3.70	0.82
Lecturers support^a^	8,915		3.47	0.78
University engagement^a^	8,315		2.31	0.89

SD, standard deviation; ^a^1 to 5, higher score indicates higher level of satisfaction with variable.

The percentages of students who reported feeling lonely during studies are shown in **Table 3**. During their time at university, 28.2% of students felt lonely. Prevalences of loneliness during studies according to several sociodemographic and other characteristics are shown in **Table 4**. We found that the percentage of lonely students was highest among gender-diverse students (45.8%), students aged 26-40 (66.5%) and those with a severely or rather affected overall health state (41.9%). Students who did not perceive culture of open communication at university reported loneliness more often (40.7%) than those with a positive perception (23.6%). In addition, our results revealed that students who felt strongly connected to their university reported lower levels of loneliness during their studies (19.6%) than those who felt only weakly connected (38.1%). Moreover, the proportion of lonely students at universities of applied sciences (20.9%) was slightly lower than at universities (29.6%).

**Table 3:** Perceived loneliness during studies (n = 8,437).

‘I am lonely in my studies’		
Response options	n	%
(1) strongly disagree	2,095	24.8
(2) disagree	2,236	26.5
(3) neither disagree, nor agree	1,724	20.5
(4) agree	1,335	15.8
(5) strongly agree	1,047	12.4
Loneliness during studies		
Perceived loneliness during studies (4-5)	2,382	28.2
No perceived loneliness during studies (1-3)	6,055	71.8

**Table 4:** Prevalence of perceived loneliness during studies by sociodemographic and other factors.

Loneliness during studies	n	%*
Gender (n = 8,162)		
Female	1,405	26.8
Male	835	30.0
Diverse	60	45.8
Age (n = 8,242)		
18 – 25	1,555	26.4
26 – 30	543	33.6
31 – 40	193	32.9
41 and older	34	24.6
Overall health state (n = 8,227)		
(Rather) not affected	541	16.9
Neither affected nor unaffected	566	26.9
Severely/rather affected	1,214	41.9
University culture of open communication (n = 7,268)		
Positive perception	1,120	23.6
Neutral perception	551	31.6
Negative perception	310	40.7
University connectedness (n = 8,114)		
(Strong) connectedness	535	19.6
Neutral connectedness	424	23.0
Weak connectedness	1,346	38.1
Type of university (n = 8,437)		
University	2,106	29.6
University of applied sciences	226	20.9

*The percentages are calculated based on students who responded both to the loneliness item and to the other corresponding items.

**Table 5** shows the results of the hierarchical logistic regression analysis to determine the associations of individual, inter-personal, and university factors with loneliness. The results of the final model are presented in the following. At the individual level, the odds of experiencing loneliness during studies were higher for gender-diverse students (OR = 1.69; 95% CI: 1.04 – 2.73) and for males (OR = 1.43; 95% CI: 1.26 – 1.63), compared with females. Additionally, students with a neutral or a poor overall health state showed higher odds of loneliness (OR = 1.56; 95% CI: 1.33 – 1.84 and OR = 2.62; 95% CI: 2.26 – 3.05, respectively) than students with a good overall health state. At the inter-personal level, we found students with a higher level of social support by their fellow students being at lower risk of reporting loneliness during their studies (OR = 0.31; 95% CI: 0.29 – 0.34). Support from lecturers was not a significant factor associated with loneliness in our analysis. At university level, we found no association between a university’s culture of open communication and loneliness. Our findings revealed that students with weak connectedness to their university were almost one and a half times more likely to report loneliness during their studies (OR = 1.43; 95% CI: 1.23 – 1.67). In addition, university engagement was associated with a lower chance of feeling lonely during studies (OR = 0.90; 95% CI: 0.83 – 0.97). A university culture of open communication was not associated with loneliness. Students who studied at a university of applied sciences were less likely to report loneliness (OR = 0.76; 95% CI: 0.63 – 0.91).

**Table 5:** Results from the hierarchical logistic regression analyses.

Domain	Variables		Loneliness at university					
			Model 1 (n = 6,655)		Model 2 (n = 6,665)		Model 3 (n = 6,665)	
			OR	95% CI	OR	95% CI	OR	95% CI
Individual level	Gender	Female (ref.)	1.00		1.00		1.00	
		Male	1.40	(1.24 – 1.58)	1.42	(1.24 – 1.61)	1.43	(1.26 – 1.63)
		Diverse	1.65	(1.07 – 2.53)	1.60	(1.00 – 2.59)	1.69	(1.04 – 2.73)
	Age	18-25 (ref.)	1.00		1.00		1.00	
		26-30	1.23	(1.07 – 1.41)	1.06	(0.91 – 1.24)	1.07	(0.92 – 1.25)
		31-40	1.21	(0.98 – 1.50)	0.97	(0.77 – 1.23)	1.02	(0.81 – 1.29)
		41 and older	0.82	(0.52 – 1.30)	0.71	(0.43 – 1.19)	0.77	(0.46 – 1.29)
	Overall health state	(Rather) not affected (ref.)	1.00		1.00		1.00	
		Neither affected nor unaffected	1.87	(1.60 – 2.17)	1.55	(1.32 – 1.82)	1.56	(1.33 – 1.84)
		Severely/rather affected	3.62	(3.16 – 4.15)	2.64	(2.27 – 3.06)	2.62	(2.26 – 3.05)
Inter-personal level	Social support among students				0.30	(0.28 – 0.33)	0.31	(0.29 – 0.34)
	Lecturers support				1.03	(0.95 – 1.12)	1.08	(0.99 – 1.18)
University level	University culture of open communication	Positive perception (ref.)					1.00	
		Neutral perception					0.92	(0.80 – 1.07)
		Negative perception					0.99	(0.80 – 1.23)
	University connectedness	(Strong) connectedness (ref.)					1.00	
		Neutral connectedness					0.93	(0.78 – 1.11)
		Weak connectedness					1.43	(1.23 – 1.67)
	University engagement						0.90	(0.83 – 0.97)
	Type of university	University (ref.)					1.00	
		University of applied sciences					0.76	(0.63 – 0.91)

OR, Odds Ratio; CI, Confidence Interval; (ref.), Reference category.

## 4 Discussion

Our study investigated the factors associated with loneliness among students in the university context based on the social-ecological framework. To the best of our knowledge, this is the first study to examine the prevalence of loneliness and the association of individual and several university characteristics with loneliness among university students, thus comparing the relative importance of individual and socio-environmental predictors.

With respect to the first aim of the study to assess the prevalence of loneliness, we found that 28.2% of students experienced loneliness, not necessarily in their everyday life, but specifically during their studies. This is in line with other research (4, 5). Thus, our study adds to prior evidence that mainly investigated general feelings of loneliness. It appears that even when COVID-19 restrictions were loosened, students were at high risk of experiencing loneliness. During the pandemic, there was a decrease in time spent socializing and a lack of interaction between students (18, 40). Especially in the aftermath of the pandemic, universities should once again be a place of common learning and social interaction.

With respect to the second aim of our study to study factors associated with loneliness we identified diverse gender identity, male gender identity, and a poor overall health state as consistent predictors of loneliness at the individual level. At the inter-personal level, higher levels of social support among students were associated with a lower likelihood of experiencing loneliness. At the organizational, i.e., university, level, students reporting weak connectedness to the university showed higher odds of experiencing loneliness during studies, while a high level of university engagement was negatively associated with loneliness. The type of university was also an associated factor – studying at a university of applied sciences was associated with a lower likelihood of experiencing loneliness.

While females were shown to be more affected by loneliness in previous works (17, 18, 26), this was not the case in our sample. We found that male and gender-diverse students were more likely to experience loneliness in their studies. However, the gender aspect of loneliness has not been conclusively established, and there are still mixed findings (5, 15, 16). There is indication that women are able to build stronger social networks at university than men (41). As Barthauer et al. (41) also noted, women could benefit from mentoring programs at universities to build their social support networks. Recently, male students showed a higher increase in loneliness than female students (4). Moreover, work by Hajek et al. (19) found associations between gender-diverse identity and loneliness, with a high prevalence of loneliness among individuals identifying themselves as gender-diverse. Our findings expand on previous research showing that gender-diverse youth is particularly susceptible to mental health issues or problems (42, 43). In our sample, they are also more likely to feel lonely than either male or female students. Further, age was not associated with loneliness. However, research in this area has also been inconsistent (4, 5), suggesting that loneliness interventions are equally important for different age groups.

Furthermore, as demonstrated in several previous studies, we found that a poor subjective health state was associated with loneliness (20, 22). In addition to loneliness interventions, universities should implement student health management programs to address health behaviors, particularly in the university context, and to create a health-promoting environment. There is a need for further qualitative research that delves deeper into the diversity-sensitive organization of universities to identify the specific needs of the individuals concerned.

Our results only partially confirmed our hypothesis that both student social support and lecturer support are negatively associated with loneliness, because a significant association could only be found for student social support. Findings of our study revealed that higher levels of social support among students act as a protective factor against loneliness at university, which is consistent with recent studies in the university context (18, 22, 44). Previous qualitative research has shown that social groups help students to find their place and develop an identity in an unfamiliar context (45), and university friends are the most important social group to protect against mental illness by reducing feelings of loneliness (46). Furthermore, social deficits were identified as a correlate of loneliness in Polish and American college students (26). In general, identifying as a part of a social group creates social connectedness, which is associated with better mental health and a decreased prevalence of depression (47).

However, our results are in contrast with previous research that found that a positive student-teacher relationship may have positive impact on adolescents’ mental health (27). Interestingly, recent school-based findings point in the opposite direction. In Scottish schools, support from teachers was associated with better mental health, while support from classmates was not (48). It is possible that the role of lecturers in young adults’ mental health changes over the course of their lives and lecturers’ roles for mental health may become less important. Our findings suggest that peer support seems to be more important than lecturer support in relation to experienced loneliness in the university context. As recently noted by McIntyre et al. (46) the campus environment provides opportunities for students to make valuable social connections, which can promote mental well-being. Universities should improve mental health support by raising awareness of social groups and organizing events to foster communities of interest.

At university level, we were able to partially confirm our hypothesis. We found that students with a weak connectedness to their university were almost one and a half times more likely to report loneliness than students with a strong connectedness. Similarly, school-based research confirmed that school inclusion had a protective effect on adolescent feelings of loneliness (33). In addition, students’ mental health may be positively influenced by a sense of belonging to the university (32, 49). In light of these results, it is noteworthy that higher levels of adjustment to university life and attachment to their university were also found among students who reported strong bonds with their peers (50).

Moreover, our findings suggest that university engagement in terms of engaging in course-related interests and participating in university activities such as general student committees and university groups are linked to a decreased likelihood of experiencing loneliness during university years. Our results confirm the assumptions that extracurricular activities are related with lower levels of perceived loneliness at university (34). However, there is previous evidence that students’ engagement with the university environment is not a predictor of mental health (32) and that loneliness is associated with mental health (5, 8, 15). Conversely, our results showed that a university culture of open communication was not associated with feelings of loneliness. In terms of mental health outcomes, previous school-based research has shown similar findings (27). Nevertheless, university culture can contribute to students’ sense of belonging (27), so both university communication and culture should be transparent and inclusive. In fact, further research is needed to explore the nuanced associations of students’ university connectedness and extracurricular engagement with loneliness. Longitudinal studies and qualitative research could provide valuable insights into these dynamics. Moreover, examining potential moderating factors such as sociodemographics and coping strategies would enhance our understanding.

Additionally, students at universities of applied sciences were less likely to report loneliness during studies than students at universities. Unlike universities, universities of applied sciences tend to have more practical teaching, smaller learning groups, and more hours on campus. These structural differences between universities and universities of applied sciences may explain the lower level of loneliness in the latter one. As universities of applied sciences tend to have smaller learning groups, our findings are consistent with school-based research suggesting that school size has little effect on pupils’ mental health (51). Nevertheless, universities should seek to provide opportunities for students to learn and interact in smaller, more consistent groups, thereby enhancing the overall campus experience.

Our study is not without limitations. Firstly, no causal claims can be made due to the cross-sectional design. However, by following a hierarchical approach to testing social-ecological domains, this allows for a detailed analysis of the comparative relevance of different predictors and their interactions, providing a differentiated insight into the relationship between variables. Future research should seek to examine changes in student loneliness over time in relation to both inter-personal and university factors. Secondly, the StudiBiFra study used a convenience sample. Therefore, selection bias cannot be ruled out, and females, those studying health subjects, and those with better health may be over-represented. This may have affected levels of university loneliness and self-rated overall health state, and an under-estimation of the prevalence of loneliness is likely. In addition, due to the stigmatized nature of loneliness, direct measures of loneliness may be prone to under-reporting (52). Furthermore, the measure of loneliness in our study should be interpreted with caution, as we explicitly asked about loneliness during studies. Rather, it represents loneliness in a particular setting. Additionally, it’s worth noting that other studies have also adopted a similar approach, focusing on loneliness within specific contexts (28, 39) and using one single item measure for loneliness is common (4, 53). Further to this, as there is no validated measure of youth loneliness (54), a single-item measure focusing on loneliness at university would be preferable to a scale that does not capture all aspects of loneliness experienced by young people. In addition, our sample had a high proportion of female participants, which may have led to gender imbalance. However, the impact of certain types of selection bias on the associations presented can be considered small, as the analysis was adjusted for gender and other factors. In addition, we were able to reach a large number of students, improving the accuracy of our estimates. We also have large geographical coverage in our sample, as we included seven universities from five federal states in Germany. Thirdly, we cannot conclusively ascertain the absence of any remaining COVID-19-related restrictions within the university settings, even though the data collection took place at a time when face-to-face teaching was generally permitted again. However, some of the lighter COVID-19 measures may still have influenced university life (e.g., wearing face masks). Further, some of our variables did not include temporal information. This may have affected factors at the inter-personal and university level because the time frame of the questions was not clearly defined, and thus previous COVID-19 measures may have influenced responses. Our results should therefore be interpreted with some caution.

## 5 Conclusions

To our knowledge, this is one of the first studies to provide evidence on factors associated with loneliness at individual, inter-personal, and organizational levels among university students at the same time. Our findings underscore the multifaceted nature of loneliness among university students. We found high levels of loneliness at universities that highlight the need for interventions targeting individual, inter-personal, and organizational factors for a supportive social campus environment. Our results reinforce the importance of positive social support among students to reduce and prevent loneliness. Therefore, universities should offer opportunities for student networking, peer-to-peer and buddy programs, with particular attention given to specific groups (e.g., gender-diverse students) in these initiatives. Findings of the present study also highlight the importance of university connectedness-based prevention measures and interventions aiming at promoting mental health and wellbeing in university settings. Promoting social connections and strengthening students’ ties to their university are important strategies for tackling loneliness, highlighting the importance of community-building initiatives. Overall, universities should strive to function as vibrant and supportive communities.

## Data Availability

The raw data supporting the conclusions of this article will be made available by the authors, without undue reservation.

## References

[1] Auerbach RP Alonso J Axinn WG Cuijpers P Ebert DD Green JG. Mental disorders among college students in the world health organization world mental health surveys. psychol Med. (2016) 46:2955–70. doi: 10.1017/s0033291716001665, PMID: 27484622 PMC5129654

[2] Heumann E Palacio Siebe AV Stock C Heinrichs K. Depressive symptoms among higher education students in Germany—a systematic review and meta-analysis. Public Health Rev. (2024) 45:1–15. doi: 10.3389/phrs.2024.1606983, PMID: 38978768 PMC11228579

[3] Heinrichs K Lehnchen J Burian J Deptolla Z Heumann E Helmer S. Mental and physical well-being among students in Germany: results from the StudiBiFra study. J Public Health. (2024). doi: 10.1007/s10389-024-02348-2

[4] Hysing M Petrie KJ Bøe T Lønning KJ Sivertsen B. Only the lonely: A study of loneliness among university students in Norway. Clin Psychol Europe. (2020) 2:1–16. doi: 10.32872/cpe.v2i1.2781, PMID: 36397982 PMC9645485

[5] Wenig V Heumann E Stock C Busse H Negash S Pischke CR. Associations of loneliness with mental health and with social and physical activity among university students in Germany: results of the Covid-19 German student well-being study (C19 Gsws). Front Public Health. (2023) 11:1284460. doi: 10.3389/fpubh.2023.1284460, PMID: 38026349 PMC10668152

[6] Zahedi H Sahebihagh MH Sarbakhsh P. The magnitude of loneliness and associated risk factors among university students: A cross-sectional study. Iran J Psychiatry. (2022) 17:411–7. doi: 10.18502/ijps.v17i4.10690, PMID: 36817808 PMC9922349

[7] Leigh-Hunt N Bagguley D Bash K Turner V Turnbull S Valtorta N. An overview of systematic reviews on the public health consequences of social isolation and loneliness. Public Health. (2017) 152:157–71. doi: 10.1016/j.puhe.2017.07.035, PMID: 28915435

[8] Beutel ME Klein EM Brähler E Reiner I Jünger C Michal M. Loneliness in the general population: prevalence, determinants and relations to mental health. BMC Psychiatry. (2017) 17:97. doi: 10.1186/s12888-017-1262-x, PMID: 28320380 PMC5359916

[9] Mizani H Cahyadi A Hendryadi H Salamah S Retno Sari S. Loneliness, student engagement, and academic achievement during emergency remote teaching during Covid-19: the role of the god locus of control. Humanities Soc Sci Commun. (2022) 9:305. doi: 10.1057/s41599-022-01328-9, PMID: 36105275 PMC9461404

[10] Duffy A Keown-Stoneman C Goodday S Horrocks J Lowe M King N. Predictors of mental health and academic outcomes in first-year university students: identifying prevention and early-intervention targets. BJPsych Open. (2020) 6:e46. doi: 10.1192/bjo.2020.24, PMID: 32381150 PMC7331085

[11] Holt-Lunstad J. Why social relationships are important for physical health: A systems approach to understanding and modifying risk and protection. Annu Rev Psychol. (2018) 69:437–58. doi: 10.1146/annurev-psych-122216-011902, PMID: 29035688

[12] Marquez J Goodfellow C Hardoon D Inchley J Leyland AH Qualter P. Loneliness in young people: A multilevel exploration of social ecological influences and geographic variation. J Public Health. (2022) 45:109–17. doi: 10.1093/pubmed/fdab402, PMID: 34999845 PMC10017088

[13] Golden TL Wendel ML. Public health’s next step in advancing equity: re-evaluating epistemological assumptions to move social determinants from theory to practice. Front Public Health. (2020) 8:131. doi: 10.3389/fpubh.2020.00131, PMID: 32457863 PMC7221057

[14] Krug EG Mercy JA Dahlberg LL Zwi AB. The world report on violence and health. Lancet. (2002) 360:1083–8. doi: 10.1016/S0140-6736(02)11133-0, PMID: 12384003

[15] Werner AM Tibubos AN Mülder LM Reichel JL Schäfer M Heller S. The impact of lockdown stress and loneliness during the Covid-19 pandemic on mental health among university students in Germany. Sci Rep. (2021) 11:22637. doi: 10.1038/s41598-021-02024-5, PMID: 34811422 PMC8609027

[16] Diehl K Hilger J. Physical activity and the transition from school to university: A cross-sectional survey among university students in Germany. Sci Sports. (2016) 31:223–6. doi: 10.1016/j.scispo.2016.04.012

[17] Labrague LJ De los Santos JAA Falguera CC. Social and emotional loneliness among college students during the Covid-19 pandemic: the predictive role of coping behaviors, social support, and personal resilience. Perspect Psychiatr Care. (2021) 57:1578–84. doi: 10.1111/ppc.12721, PMID: 33410143

[18] Elmer T Mepham K Stadtfeld C. Students under Lockdown: Comparisons of Students’ Social Networks and Mental Health before and During the Covid-19 Crisis in Switzerland. PLoS One. (2020) 15:e0236337. doi: 10.1371/journal.pone.0236337, PMID: 32702065 PMC7377438

[19] Hajek A König HH Blessmann M Grupp K. Loneliness and social isolation among transgender and gender diverse people. Healthcare (Basel). (2023) 11:1–10. doi: 10.3390/healthcare11101517, PMID: 37239802 PMC10217806

[20] Christiansen J Qualter P Friis K Pedersen SS Lund R Andersen CM. Associations of loneliness and social isolation with physical and mental health among adolescents and young adults. Perspect Public Health. (2021) 141:226–36. doi: 10.1177/17579139211016077, PMID: 34148462

[21] Matthews T Danese A Caspi A Fisher HL Goldman-Mellor S Kepa A. Lonely young adults in modern Britain: findings from an epidemiological cohort study. Psychol Med. (2019) 49:268–77. doi: 10.1017/s0033291718000788, PMID: 29684289 PMC6076992

[22] Peltzer K Pengpid S. Loneliness: its correlates and associations with health risk behaviours among university students in 25 countries. J Psychol Afr. (2017) 27:247–55. doi: 10.1080/14330237.2017.1321851

[23] Hurst CS Baranik LE Daniel F. College student stressors: A review of the qualitative research. Stress Health. (2013) 29:275–85. doi: 10.1002/smi.2465, PMID: 23023893

[24] Holt-Lunstad J. The major health implications of social connection. Curr Dir psychol Sci. (2021) 30:251–9. doi: 10.1177/0963721421999630

[25] Holt-Lunstad J Robles TF Sbarra DA. Advancing social connection as a public health priority in the United States. Am Psychol. (2017) 72:517–30. doi: 10.1037/amp0000103, PMID: 28880099 PMC5598785

[26] Hopmeyer A Terino B Adamczyk K Corbitt Hall D DeCoste K Troop-Gordon W. The lonely collegiate: an examination of the reasons for loneliness among emerging adults in college in the western United States and west-central Poland. Emerging Adulthood. (2022) 10:237–48. doi: 10.1177/2167696820966110

[27] Long E Zucca C Sweeting H. School climate, peer relationships, and adolescent mental health: A social ecological perspective. Youth Soc. (2021) 53:1400–15. doi: 10.1177/0044118x20970232, PMID: 34848899 PMC7612050

[28] Schnepf SV Boldrini M Blaskó Z. Adolescents’ Loneliness in European schools: A multilevel exploration of school environment and individual factors. BMC Public Health. (2023) 23:1917. doi: 10.1186/s12889-023-16797-z, PMID: 37794392 PMC10548635

[29] Jefferson R Barreto M Jones F Conway J Chohan A Madsen KR. Adolescent loneliness across the world and its relation to school climate, national culture and academic performance. Br J Educ Psychol. (2023) 93(4):997–1016. doi: 10.1111/bjep.12616, PMID: 37248510

[30] Heumann E Trümmler J Stock C Helmer SM Busse H Negash S. Study conditions and university students’ mental health during the pandemic: results of the Covid-19 German student well-being study (C19 Gsws). Int J Environ Res Public Health. (2023) 20:5286. doi: 10.3390/ijerph20075286, PMID: 37047902 PMC10094523

[31] Matos Fialho PM Spatafora F Kühne L Busse H Helmer SM Zeeb H. Perceptions of study conditions and depressive symptoms during the Covid-19 pandemic among university students in Germany: results of the international Covid-19 student well-being study. Front Public Health. (2021) 9:674665. doi: 10.3389/fpubh.2021.674665, PMID: 34178930 PMC8222519

[32] Fink JE. Flourishing: exploring predictors of mental health within the college environment. J Am Coll Health. (2014) 62:380–8. doi: 10.1080/07448481.2014.917647, PMID: 24779485

[33] Arslan G. School belongingness, well-being, and mental health among adolescents: exploring the role of loneliness. Aust J Psychol. (2021) 73:70–80. doi: 10.1080/00049530.2021.1904499

[34] Bone JK Fancourt D Fluharty ME Paul E Sonke JK Bu F. Cross-sectional and longitudinal associations between arts engagement, loneliness, and social support in adolescence. Soc Psychiatry Psychiatr Epidemiol. (2023) 58:931–8. doi: 10.1007/s00127-022-02379-8, PMID: 36342533 PMC10241709

[35] Jefferson R Barreto M Verity L Qualter P. Loneliness during the school years: how it affects learning and how schools can help(). J Sch Health. (2023) 93:428–35. doi: 10.1111/josh.13306, PMID: 36861756

[36] Schaefer DR Simpkins SD Vest AE Price CD. The contribution of extracurricular activities to adolescent friendships: new insights through social network analysis. Dev Psychol. (2011) 47:1141–52. doi: 10.1037/a0024091, PMID: 21639618 PMC3134619

[37] Buecker S Ebert T Götz FM Entringer TM Luhmann M. In a lonely place: investigating regional differences in loneliness. Soc psychol Pers Sci. (2021) 12:147–55. doi: 10.1177/1948550620912881

[38] Lehnchen J Helmer SM Heinrichs K Burian J Deptolla Z Heumann E. Assessment of study conditions, needs for action and students’ mental health in Germany – results from the cross-sectional StudiBiFra study. J Public Health. (2025). doi: 10.1007/s10389-024-02387-9

[39] Ladd GW Ettekal I. Peer-related loneliness across early to late adolescence: normative trends, intra-individual trajectories, and links with depressive symptoms. J Adolescence. (2013) 36:1269–82. doi: 10.1016/j.adolescence.2013.05.004, PMID: 23787076

[40] Giuntella O Hyde K Saccardo S Sadoff S. Lifestyle and mental health disruptions during Covid-19. Proc Natl Acad Sci U S A. (2021) 118:1–9. doi: 10.1073/pnas.2016632118, PMID: 33571107 PMC7936339

[41] Barthauer L Spurk D Kauffeld S. Women’s social capital in academia: A personal network analysis. Int Rev Soc Res. (2016) 6:207–32. doi: 10.1515/irsr-2016-0022

[42] Wittlin NM Kuper LE Olson KR. Mental health of transgender and gender diverse youth. Annu Rev Clin Psychol. (2023) 19:207–32. doi: 10.1146/annurev-clinpsy-072220-020326, PMID: 36608332 PMC9936952

[43] Newcomb ME Hill R Buehler K Ryan DT Whitton SW Mustanski B. High burden of mental health problems, substance use, violence, and related psychosocial factors in transgender, non-binary, and gender diverse youth and young adults. Arch Sex Behav. (2020) 49:645–59. doi: 10.1007/s10508-019-01533-9, PMID: 31485801 PMC7018588

[44] Backhaus I Fitri M Esfahani M Ngo HT Lin LJ Yamanaka A. Mental health, loneliness, and social support among undergraduate students: A multinational study in Asia. Asia Pac J Public Health. (2023) 35:244–50. doi: 10.1177/10105395231172311, PMID: 37226778

[45] Maunder RE Cunliffe M Galvin J Mjali S Rogers J. Listening to student voices: student researchers exploring undergraduate experiences of university transition. Higher Educ. (2013) 66:139–52. doi: 10.1007/s10734-012-9595-3

[46] McIntyre JC Worsley J Corcoran R Harrison Woods P Bentall RP. Academic and non-academic predictors of student psychological distress: the role of social identity and loneliness. J Ment Health. (2018) 27:230–9. doi: 10.1080/09638237.2018.1437608, PMID: 29436883

[47] Lund C Brooke-Sumner C Baingana F Baron EC Breuer E Chandra P. Social determinants of mental disorders and the sustainable development goals: A systematic review of reviews. Lancet Psychiatry. (2018) 5:357–69. doi: 10.1016/S2215-0366(18)30060-9, PMID: 29580610

[48] Goodfellow C Hardoon D Inchley J Leyland AH Qualter P Simpson SA. Loneliness and personal well-being in young people: moderating effects of individual, interpersonal, and community factors. J Adolescence. (2022) 94:554–68. doi: 10.1002/jad.12046, PMID: 35403218 PMC9320932

[49] Santini ZI Pisinger VSC Nielsen L Madsen KR Nelausen MK Koyanagi A. Social disconnectedness, loneliness, and mental health among adolescents in Danish high schools: A nationwide cross-sectional study. Front Behav Neurosci. (2021) 15:632906. doi: 10.3389/fnbeh.2021.632906, PMID: 33927599 PMC8078177

[50] Maunder RE. Students’ Peer relationships and their contribution to university adjustment: the need to belong in the university community. J Further Higher Educ. (2018) 42:756–68. doi: 10.1080/0309877X.2017.1311996

[51] Patalay P O’Neill E Deighton J Fink E. School characteristics and children’s mental health: A linked survey-administrative data study. Prev Med. (2020) 141:106292. doi: 10.1016/j.ypmed.2020.106292, PMID: 33075351

[52] Shiovitz-Ezra S Ayalon L. Use of direct versus indirect approaches to measure loneliness in later life. Res Aging. (2012) 34:572–91. doi: 10.1177/0164027511423258

[53] Shiovitz-Ezra S Ayalon L. Situational versus chronic loneliness as risk factors for all-cause mortality. Int Psychogeriatrics. (2010) 22:455–62. doi: 10.1017/S1041610209991426, PMID: 20003631

[54] Qualter P Verity L Walibhai W Fuhrmann D Riddleston L Alam I. Exploring child and youth understanding of loneliness through qualitative insights and evaluating loneliness measures considering those lived experiences. Ann New York Acad Sci. (2025) 1544(1):42–54. doi: 10.1111/nyas.15269, PMID: 39873211 PMC11829323

